# Deriving Real-World Evidence from Non-English Electronic Medical Records in Hormone Receptor-Positive Breast Cancer Using Large Language Models

**DOI:** 10.3390/cancers17233836

**Published:** 2025-11-29

**Authors:** Daur Meretukov, Katerina Grechukhina, Vladimir Evdokimov, Dmitry Didych, Sofia Kondratieva, Olga Rakitina, Alexander Gordeev, Polina Shilo, Igor Khatkov, Lyudmila Zhukova

**Affiliations:** 1 Department of Science, N.N. Blokhin Cancer Research Center, Moscow 115478, Russia; 2 SBIH Moscow Clinical Scientific Center Named After A.S. Loginov of DHM, Moscow 111123, Russia; dr.grechukhina@gmail.com (K.G.); i.hatkov@mknc.ru (I.K.); zhukova.lyudmila008@gmail.com (L.Z.); 3 SBIH Moscow Multidisciplinary Clinical Center “Kommunarka” of DHM, Moscow 117638, Russia; evdokimov.onco@gmail.com; 4 Group of Gene Immuno-Oncotherapy, Department of Genomics and Postgenomic Technologies, Shemyakin-Ovchinnikov Institute of Bioorganic Chemistry of the Russian Academy of Sciences, Moscow 117997, Russia; dmitrydid@gmail.com (D.D.); rakitinaolga97@gmail.com (O.R.); 5 LLC “Technology of Trust”, Moscow 125167, Russia; a8gordeev@gmail.com; 6 Lahta Clinic Medical Center, St. Petersburg 197183, Russia; polinashilo0@gmail.com

**Keywords:** large language model, LLM, breast cancer, BC, “luminal B poor-prognosis” breast cancer, LPP, real-world data, RWD, artificial intelligence, AI

## Abstract

**Background/Objectives:** Large language models (LLMs) have been proposed as a means of converting unstructured electronic medical records (EMRs) into structured datasets. However, concerns regarding the reliability of these models in non-English clinical text and their capacity to generate novel insights remain unresolved. We aimed to utilize an LLM to identify a hypothetical “Luminal B poor-prognosis” breast cancer subgroup (LPP) based on progesterone receptor (PR), the Ki-67 proliferation index, and grade characteristics, while concurrently validating the LLM’s accuracy. **Methods:** We retrospectively compiled the EMRs on 7756 female breast cancer patients from five Moscow oncology centers. An LLM with a domain-engineered prompt extracted eight clinicopathological variables (Ki-67, estrogen receptor (ER)/PR Allred status, HER2 status, grade, relapse dates, and multiple primaries). The accuracy of the model was validated in 366 randomly sampled cases against oncologist annotations using Intraclass Correlation Coefficient (ICC) and weighted κ. Following data post-processing, the complete-case cohort (*n* = 2347) and the HR+/HER2− stage I–III sub-cohort (*n* = 1419) were analyzed. Survival was estimated with Kaplan–Meier/log-rank and modeled with Cox regression (adjusted for age, stage, and treatment). Ki-67 was modeled continuously; prespecified LPP definitions were compared. **Results:** LLM–human agreement was high (Ki-67 ICC = 0.882; grade κ = 0.887; ER κ = 0.997; PR κ = 0.975; HER2 κ = 0.935). Date extraction was characterized by a high degree of missing data. In HR+/HER2− stage I–III disease, ER < 5 was non-prognostic; however, PR < 4 and Ki-67 ≥ 40% were indicative of inferior survival (HR 2.25 and 1.85). The most effective LPP definition (PR < 4 and Ki-67 ≥ 40%) identified a subgroup (~5.3%) of patients with markedly poorer outcomes (age, stage, and treatment adjusted HR 2.60, 95% CI 1.53–4.43) compared to the Luminal B (HER2−) subgroup. **Conclusions:** The developed LLM has demonstrated the ability to reliably structure non-English EMRs and enable discovery of clinically meaningful subgroups. The discovered LPP phenotype defines a small, high-risk subset warranting external validation. Given the retrospective, single-system design of the study, it is imperative to interpret the discovered phenotype features as hypothesis-generating, rather than as definitive evidence.

## Simple Summary

Electronic medical records (EMRs) contain valuable clinical information, but a portion of this data is recorded in free-text, making it difficult to analyze. Large language models (LLMs) are a type of artificial intelligence tool that can transform the unstructured notes into structured data, which can be useful for research purposes. We have accessed the capacity of an LLM to accurately process Russian breast cancer EMRs and conducted a subsequent analysis of the data to identify novel clinical insights. The model demonstrated the capacity to accurately extract key tumor features, including hormone receptor status, HER2 status, and tumor grade, exhibiting a high degree of agreement with the assessment of the same data by expert oncologists. Using the structured dataset, we found that patients exhibiting both very low progesterone receptor (PR) levels and very high tumor cell growth (Ki-67 ≥ 40%) had significantly worse outcomes than other luminal breast cancers. We refer to this small, high-risk group as the “Luminal B poor-prognosis” (LPP) subtype. While these findings are hypothesis-generating, identifying the LPP subtype may help refine treatment decisions in the future, subject to external validation and prospective testing.

## 1. Introduction

Breast cancer (BC) is the most prevalent cancer type among female patients and a major contributor to cancer-related mortality worldwide [1]. The majority of BC cases is represented by human epidermal growth factor receptor 2 (HER2)-negative, hormone receptor (HR, estrogen (ER) and progesterone (PR) receptor)-positive (HR+/HER2−) tumors. Patients with this BC subtype are usually diagnosed with stages I–III of the disease. Up to 30% of patients relapse, often within the first few years, despite generally favorable long-term outcomes [2,3,4]. This clinical heterogeneity highlights the need for better risk stratification that goes beyond conventional prognostic markers. Prognosis is significantly influenced by well-established clinicopathological factors, such as nodal status, tumor grade, Ki-67 proliferation index, and progesterone receptor expression on immunohistochemistry (IHC) [5,6,7,8]. HR+/HER2− cancers are typically classified into two subtypes: Luminal A and Luminal B. Luminal A is associated with favorable outcomes and reduced proliferation index, whereas Luminal B is characterized by a higher risk of recurrence and proliferation index. However, significant variations in outcomes have been observed even within the Luminal B subtype: while some tumors exhibit aggressive characteristics, akin to those observed in the triple-negative BC type, the others maintain sensitivity to endocrine therapy [4,9,10]. This aggressive phenotype has molecular correlates, according to recent evidence. These correlates include low or absent ER/PR expression, high Ki-67 proliferation index, and higher grade. In example, low or absent PR expression—especially <20% or <4 on the Allred scale—is consistently associated with worse outcomes, with hazard ratios for disease-free survival ranging from 1.48 to 2.70 [11,12,13]. Additionally, it was also shown that low ER status (0 to 5 on the Allred scale) was associated with poor outcomes [14]. Similarly, early metastasis and recurrence are predicted by high Ki-67 (>20–25%) [15,16,17]. A particularly high-risk group is defined by low PR and elevated Ki-67, which can triple the risk of recurrence. This profile is further refined by tumor grade: grade 3 tumors are strongly associated with aggressive Luminal B phenotype and frequently exhibit Ki-67 values of about 40%, rather than about 10% in grade 1 tumors [18,19,20]. Together, these findings suggest the presence of a distinct poor-prognosis phenotype within HR+/HER2− breast cancer, which can be distinguished by three overlapping characteristics, high proliferation index (Ki-67 > 25%), poor tumor differentiation (grade 3), and low PR expression (<20% or <4 Allred score), which is hereafter referred to as the “Luminal B poor-prognosis” (LPP) phenotype. Identifying this subgroup is hypothesis-generating and may inform future risk stratification in clinical decision-making after proper external validation. The advancement of this field has been hindered by limitations inherent in real-world data. The generation of clinical databases is often performed manually from unstructured medical records, thus being a laborious, error-prone, and challenging process to scale [21,22]. A potential remedy to this problem is provided by recent developments in large language models (LLMs), which today achieve state-of-the-art performance in various natural language processing tasks, including entity recognition, data extraction, and clinical text summarization [23,24,25,26]. By facilitating automated, high-throughput analysis of electronic medical records, these tools enable the exploration of new concepts and the validation of established prognostic markers. Nevertheless, the generalizability of the majority of LLM evaluations is restricted by the use of purely English-based corpora. Benchmarking studies continue to under-represent clinical texts written in languages other than English, such as Spanish, Chinese, or Russian [27,28]. In light of the Chinese [29] and Swedish [30] medical LLM benchmarks emphasizing the value of language-specific evaluations for patient safety, recent research has begun to address this issue. The majority of LLM applications continue to utilize English-only data, even in oncology [31]. Therefore, it is crucial to extend assessments into non-English contexts to guarantee the dependability of AI-driven tools across various healthcare systems.

In this study, we validated the accuracy of LLM in extracting key variables from Russian EMR data and pathology reports for breast cancer patients, with a specific focus on HR+/HER2− stage I-III disease. We have used the extracted data to perform analyses to address the following problems:

- To verify established clinicopathological prognostic factors in a population in Moscow.
- To look into the clinico-morphological traits of the suggested LPP subgroup, with an emphasis on how patient outcomes are impacted by low PR expression, poor differentiation, and high proliferation.

## 2. Materials and Methods

### 2.1. Study Design and Data Sources

Within this retrospective, multicenter cohort study, the electronic medical records (EMRs) from five high-volume oncology centers in Moscow were utilized. The patients diagnosed with breast cancer (BC) in 2019 were included. The final cohort comprised 7756 unique patients. The survival data extraction cutoff was 28 February 2025. The following inclusion criteria for initial free-text data gathering were established:

- Female sex;
- Age ≥ 18 years at diagnosis;
- ICD-10 code C50.X recorded in the EMR;
- Date of pathologically confirmed diagnosis between 1 January 2019 to 31 December 2019;
- Non-empty target fields (“disease history”, “extended diagnosis”, “pathology reports”).

Each institution involved in this study extracts data through the same governmental EMR provider (EMIAS, Moscow, Russia) in an encrypted system without external access. All records were fully de-identified before transfer to the study team.

### 2.2. Raw-Text Dataset Construction

By official request, the EMIAS vendor provided us with a single Microsoft Excel (.xlsx) file containing 4 columns: patient ID (unique anonymized patient identifier), the most recent full patient history, extended diagnosis written by the patient’s physician, and pathology reports available for this patient. Additionally, EMIAS extracted a second .xlsx file with the following data: patient ID (similar to .xlsx with histories free-text), date of birth, date of diagnosis, TNM staging, clinical stage, date of breast cancer surgery, date of last visit, survival status, and death date. Additionally, EMIAS extracted structured coarse systemic treatment data which included neoadjuvant (NAT) or adjuvant (AT) treatment. Treatment was classified into 4 variables according to EMR fields: endocrine therapy (ET), chemotherapy (CT), combination (ET + CT) therapy, or no therapy (None). All the datasets were inner-joined by the unique patient ID. No other automated pre-processing was undertaken by EMIAS at this point. For survival analyses, surgery date, last visit date, and survival status/death date were taken as-is from these structured EMIAS fields and used for time origin/censoring or event dating where applicable.

### 2.3. Prompt Engineering and Large Language Model Extraction

As well-designed prompts remain the cornerstone of accurate LLM-based data retrieval, we have approached this task in an interdisciplinary manner. The domain-specific prompt (see Supplementary data, Supplementary File S1) was designed by two experienced board-certified medical oncologists and one IT specialist. As a part of prompt engineering, we have randomly selected an illustrative example patient case (*n* = 1), which was subsequently excluded from the validation dataset. To further improve the prompt efficacy, we enriched it with breast cancer terms and definitions (“in-prompt dictionary”), supported by synthetic examples. These examples were not drawn from validation or analysis cases.

We have selected Claude Sonnet 3.7 (Anthropic)—a reliable, state-of-the-art model that allows users to build an API-based approach to run large volumes of unstructured data with a reasonable cost-effectiveness—as a basic LLM. To reduce the hallucination rate and minimize over-extraction, we have provided positive and negative examples for each variable with an additional fully annotated patient. Additionally, we executed model calls with temperature 0 and default nucleus sampling.

In the prompt, we requested the extraction of 8 major variables related to our clinical hypothesis, with the expected formats and examples as described in Table 1.

To handle the missing and/or conflicting information, we have included the following prompt-based rules:

- Multiple primary cancers: If any text indicated an additional malignancy, both annotators (see below) and the LLM recorded “yes” for multiple cancers and left all other tumor-specific variables blank to avoid bias for the model as it might increase context complexity and model excluded patients with “yes” annotation from final dataset automatically.
- Repeated events: For local or distant progression, the earliest documented date was extracted.
- Clinically incorrect values: Out-of-range values (e.g., Ki-67 > 100 %, grade > 3, ER > 8) were retained “as written” during extraction and removed during a rule-based post-filtering step in R (see below).
- Missing values: Blanks were preserved in the final dataset generated by the LLM. During validation, a missing–missing match was scored as concordant to avoid penalizing the model for absent source data.

To run the extraction process, a custom Python 3.11 script was designed to integrate with Anthropic API with the following algorithm:

1. Extract full-text data per patient from an .xlsx raw-data document;
2. Input a designed prompt with each patient data;
3. Extract each generated JSON object with structured patient data;
4. Parse each JSON object;
5. Generate a human-readable .docx intermediate report per patient for auditability;
6. Appended the parsed values to a new Excel file.

No directly identifying information was transmitted outside the protected environment. Before any model inference, the EMR vendor (EMIAS) released a de-identified corpus to our research group under ethics approval [protocol № 4/2022, 21 April 2022]. The de-identification pipeline removed names/patronymics, national insurance (SNILS), passport numbers, phone/email, and other data. The few residual facility or city names that may appear were judged non-identifying under our approval. For LLM extraction, only this de-identified text was sent via API over encrypted channels from a controlled server. No raw identifiers or linkable keys left the protected environment.

### 2.4. Validation of LLM Extraction

From the 7756 breast cancer cases initially processed by the LLM, we have randomly selected a validation subset of 366 patients (≈5%) for AI–human comparison. This specific sample size was chosen in order to balance feasibility with statistical rigor, as all cases required manual annotation by five board-certified oncologists. Due to resource constraints, only one-tenth of patients (*n* = 37) were secondarily reviewed by a senior oncologist for quality control.

Progression dates were parsed and compared categorically (match vs. mismatch). Biomarkers were standardized to clinical scales (Grade G1–G3, HER2 0/1+/2+/3+, ER/PR 0–8, Ki-67 0–100). Missing values were handled by predefined rules: both missing = match; one missing = mismatch.

For dates: match rate and median absolute day difference. For ordinal biomarkers (Grade, HER2, ER, PR): match rate, weighted κ (kappa); for ER/PR also ±1 category agreement. Continuous (Ki-67): ICC (Intraclass Correlation Coefficient, absolute agreement), MAE (Mean Absolute Error), RMSE (Root Mean Square Error), bias, and Bland–Altman analysis with 95% confidence intervals (CI) for κ and ICC. All analyses were performed in R (2024.04.1+748).

### 2.5. Post-Processing and Data Cleaning

As a result, extraction yielded 7756 unique patient cases. There was a significant proportion of missing data in original free-text electronic medical records since not all fields were obligatory to be filled by physicians since 2019. Therefore, we filtered these cases to keep only non-empty fields for “Ki-67”, “ER”, “PR”, “Grade”, and “HER2”. The final filtered dataset for clinical analysis comprised 2347 unique cases. Minor LLM formatting artifacts were removed in R 4.4.0 (e.g., stripping “grade” suffixes from ER numeric values, standardizing “HER2 1+” to “1+”) using regular-expression rules (package stringr). We applied filtering to select patient subgroups according to our clinical hypotheses—filtered by ER, PR, and HER2+ status and simplified (from I to IV) clinical stages associated with original raw dataset TNM staging.

### 2.6. Statistical Analysis of Clinical Data

Analysis was performed both in the overall study cohort and in the subgroup of patients with HR+/HER2− BC. Baseline clinicopathologic data included age, tumor stage, histologic grade, estrogen receptor (ER), progesterone receptor (PR), HER2, and Ki-67 expression. The data cutoff was 28 February 2025. Patients without an event were censored at the last date of follow-up. Vital status was last ascertained on 28 February 2025, from structured EMIAS death status/date fields. Patients alive on or before that date were censored at their last physician contact recorded in EMIAS.

The overall survival (OS) was defined as time from date of diagnosis (structured EMR field) until death. For localized disease (stage I–III cases), disease-free survival (DFS) was defined as time from surgery date until disease relapse or death. For DFS, time origin was the date of curative breast surgery (structured EMR field obtained via EMIAS provider). The event was the earliest of (1) local or distant relapse documented in EMR text with an exact calendar date (dd.mm.yyyy), or (2) death from any cause. If both local and distant relapse dates were documented, the earliest relapse date was used. If multiple relapse dates appeared across notes, we selected the chronologically earliest exact date. Cases without an exact relapse date (e.g., month-only or relative phrases) were ignored for dating; cases remained at risk until a dated event or censoring. We did not impute relapse dates. Patients alive without a dated relapse were censored at the last recorded physician visit (structured EMR field).

For metastatic disease (stage IV), progression-free survival (PFS) was defined as time from initial treatment to recurrence. However, the PFS in metastatic patients was not modeled due to the fact that systemic therapy start dates were inconsistently available in source records. Thus, metastatic patients contributed to OS analyses only. We analyzed OS as specified below, with death status and date obtained from structured EMR fields via the EMIAS provider.

All statistical analyses were conducted using R (2024.04.1+748). All statistical tests were two-sided, and *p* < 0.05 was considered statistically significant. Survival probabilities were estimated using the Kaplan–Meier method, and differences between groups were evaluated by the log-rank test. Median survival, restricted mean survival times, and 1-, 3-, and 5-year survival estimates are reported with 95% CIs.

Associations between clinicopathologic variables and survival were evaluated using Cox proportional hazards regression models. Results are presented as hazard ratios (HRs) with corresponding 95% CIs and two-sided *p*-values. Multivariable models were adjusted for age at diagnosis, simplified clinical stage (associated T, N, M categories), and systemic therapy. The systemic treatment included neoadjuvant (NAT) or adjuvant (AT) treatment, and was classified into 4 variables according to EMR fields: endocrine therapy (ET), chemotherapy (CT), combination (ET + CT) therapy, or no therapy (None). The therapy regimen was modeled as a unified four-level covariate (CT, ET, CT + ET, None), irrespective of NAT or AT timing. This specification was chosen to capture treatment modality/intensity while avoiding sparsity and quasi-separation observed when NAT and AT regimens were modeled separately. The combined variable improved numerical stability and model performance and reflects that antitumor agents that used NAT and AT are largely identical in early HR+/HER2− disease.

### 2.7. Definition of «LPP» Subtype

We modeled Ki-67 continuously using natural cubic splines (df = 4) in Cox models adjusted for age and stage; interaction with PR (<4 vs. ≥4) was assessed by an ns (Ki-67) × PR term. Curves show HRs relative to a stratum-specific reference at 30% (see the “Results” section below.

Within the HR+/HER2− stage I–III cohort, we prespecified a set of clinicopathologic rules based on routine IHC: PR dichotomized at Allred <4 vs. ≥4 and Ki-67 at 28%, 30%, 35%, and 40%; we also evaluated the optional inclusion of grade 3. For each candidate, we created a binary label and compared OS (primary endpoint) by Kaplan–Meier/log-rank, quantified separation with univariable Cox hazards and concordance, and recorded prevalence to ensure clinical deployability. We used OS as the primary endpoint for inference, whilst the DFS analysis in stage I–III was supportive and reported with the operational rules above. The best-performing rule was then encoded into a three-level subtype variable (Luminal A, Luminal B (HER2−), LPP) and tested in multivariable Cox models adjusted for age at diagnosis, stage, and treatment regimen; model discrimination (c-index) and incremental fit (likelihood ratio test vs. a model without subtype) were reported.

### 2.8. Classification Rules for Luminal A, Luminal B (HER2−), and LPP

Classification of HR+/HER2− tumors was performed using a fully prespecified and reproducible rule set based on ER, PR (Allred), Ki-67 (%), grade, and HER2 status.

We define the LPP group as tumors meeting both criteria were classified as Luminal B poor-prognosis (LPP): PR < 4 (Allred) and Ki-67 ≥ 40%. We define Luminal A (HER2−): ER ≥ 5 (Allred), PR ≥ 4 (Allred), Ki-67 ≤ 20%, Grade 1–3, and HER2-negative. Finally, we define Luminal B (HER2−) as tumors not fulfilling Luminal A criteria and not meeting the LPP rule. In practice, this includes the following patterns: PR < 4 (Allred) with Ki-67 < 40%, Ki-67 > 20% and <40% regardless of PR, high grade (G3) with preserved PR, and any single abnormality (PR < 4 or Ki-67 ≥ 40%). The complete algorithm is summarized in Supplementary Table S1.

Discordant combinations were assigned as described in Supplementary Table S2. Cases with PR < 4 and Ki-67 ≥ 40% were classified as LPP. Other combinations (PR < 4 AND Ki-67 20–39%, PR ≥ 4 AND Ki-67 ≥ 40%, etc.) were assigned to Luminal B (HER2−).

This rule-based system ensured that LPP was the only subgroup defined strictly by a dual high-risk pattern, whereas all other HR+/HER2− tumors were objectively routed into Luminal A or Luminal B according to fixed, reproducible criteria.

## 3. Results

### 3.1. LLM Validation Results

#### 3.1.1. Ki-67 Proliferation Index

**Figure 1:**
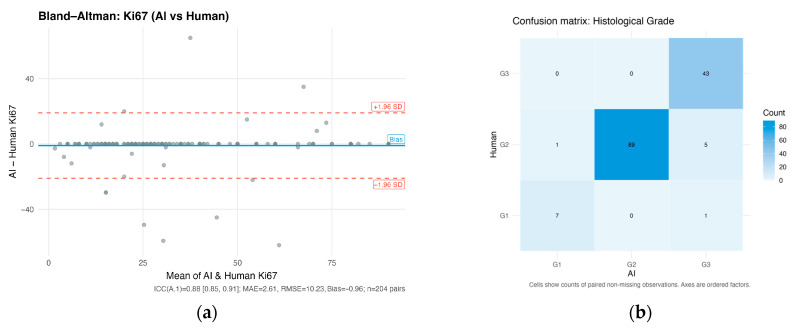

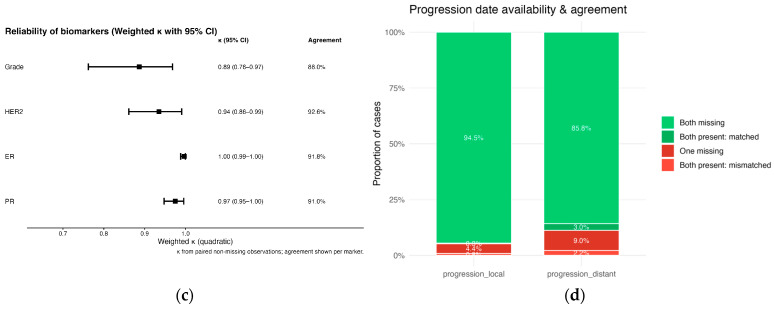
Validation of AI-assisted extraction from pathology reports. (**a**) Bland–Altman plot for Ki-67 proliferation index. Each grey spot represents one unique case. Darker gray spots are overlapping cases; (**b**) confusion matrix for histological grade; (**c**) forest plot for ER, PR, and HER2 receptor status agreement rates; (**d**) barplots illustrating progression dates availability and agreement rates.

For 204 paired values, agreement between human annotator and LLM was 85.2%. The ICC was 0.882 (95% CI: 0.85–0.91), reflecting strong reliability. Error metrics showed MAE = 2.61%, RMSE = 10.23%, and mean bias = −0.96% (AI slightly lower than human). Bland–Altman analysis demonstrated no systematic trend, with most differences clustering near zero but a few outliers showing differences of up to ±40 percentage points (Figure 1a).

#### 3.1.2. Histological Grade

Among 146 patients with both annotations, raw agreement was 88.0% with a weighted κ of 0.887 (95% CI: 0.76–0.97), indicating strong concordance between AI and human annotations with most discrepancies occurring between adjacent grades, indicating that the AI reliably distinguishes grade 2 and grade 3 cases but has occasional difficulty differentiating closely related grades (Figure 1b,c).

#### 3.1.3. Receptor Status for ER, PR, and HER2

For HER2 status in 213 paired annotations, agreement reached 92.6%, with a weighted κ of 0.935 (95% CI: 0.86–0.99), consistent with almost perfect reliability. For ER status, in 180 paired annotations, agreement was 91.8% with κ = 0.997 (95% CI: 0.99–1.00). Within the ±1 scoring category, agreement was 92.9%. PR status showed close results in 181 cases with agreement of 91.0% with κ = 0.975 (95% CI: 0.95–0.99). Within the ±1 category, agreement was 91.5% (Figure 1c).

Both markers showed near-perfect consistency between AI and human annotation.

#### 3.1.4. Progression Dates

For local progression, agreement was high (94.8%), but nearly all cases were jointly missing (346/366). Only four cases had both dates present; when mismatched, the median discrepancy was 2186 days (~6 years).

For distant progression, agreement was 88.8%, with 314 cases missing on both sides. This reflects the validation subset’s composition (predominantly I–III stage disease within ≈5-year observation) and our validation protocol that counted both missing as agreement, rather than the event rates in the analysis cohorts. Among the 19 cases with both dates present, mismatched annotations showed a median difference of 410 days (Figure 1d; Supplementary Figures S1 and S2).

### 3.2. Descriptive Statistics and Cutoff Evaluation

**Figure 2:**
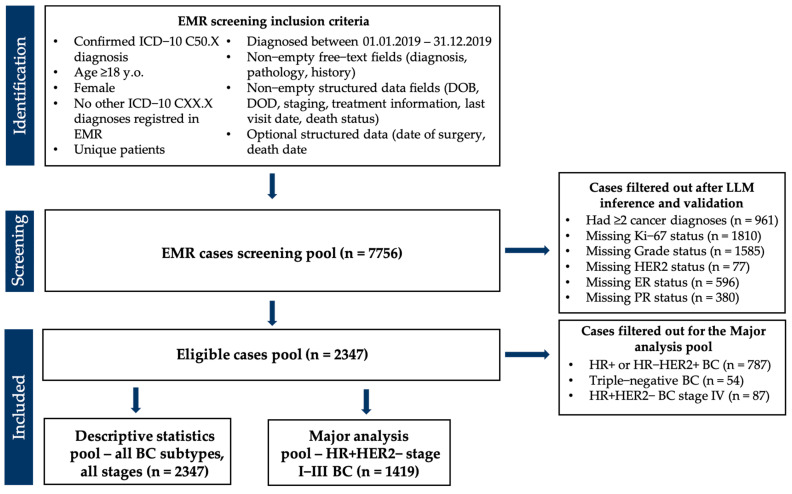
CONSORT diagram demonstrating screening cohort selection for the study and step-by-step exclusion criteria for the final cohort.

In total, 2347 patients were included in the final clinical study cohort after filtering a validated output LLM-generated structured dataset on non-empty clinicopathological information (Figure 2).

For HR+/HER2−, 1419 had non-metastatic disease at diagnosis (stage I–III). Median age at diagnosis was 60.8 years (range 23.7–95.0) in the overall cohort and 61.3 years (24.1–99.0) in the HR+HER2− cohort; median follow-up was 61.5 and 61.6 months, respectively. Overall stage distribution was I/II/III/IV: 29.9% (673)/43.2% (971)/20.2% (454)/6.7% (150); in HR+HER2−, it was I/II/III: 35.0% (497)/44.5% (631)/20.5% (291). Molecular subtypes in the overall cohort were Luminal A 17.4% (409), Luminal B (HER2−) 46.7% (1097), Luminal B (HER2+) 29.8% (698), HR–/HER2+ 3.8% (89), and triple-negative 2.4% (54); within HR+HER2− stage I–III, Luminal A and Luminal B (HER2−) comprised 27.8% (394) and 72.2% (1025). Median Ki-67 was 24% (IQR 15–35) overall and 25% (IQR 16–35) in HR+HER2−. ER (Allred) was ≥5 in 90.9% (2132) overall and 97.7% (1387) in HR+HER2−; PR (Allred) was ≥4 in 78.8% (1849) and 85.9% (1219), respectively. Histologic grade was G1/G2/G3: 8.5% (201)/67.1% (1575)/24.3% (571) overall and 10.8% (153)/69.6% (988)/19.6% (278) in HR+HER2−. During follow-up, 317 deaths (13.5%) and 629 progression/relapse events (26.8%) occurred overall, versus 130 deaths (9.2%) and 291 progression/relapse events (20.2%) in the HR+/HER2− stage I-III cohort. Additionally, the latter cohort *n* = 238 patients had a dated relapse, while *n* = 53 had death without a documented relapse (counted as events at the death date), and *n* = 1128 patients were censored at last contact.

Treatment allocation (NAT and AT combined) differed across subtypes (χ^2^ test *p* < 0.0001). Within-subtype distributions were Luminal A—CT 49/394 (12.4%), CT+ET 31/394 (7.9%), ET 303/394 (76.9%), None 11/394 (2.8%); Luminal B (HER2−)—CT 308/950 (32.4%), CT+ET 261/950 (27.5%), ET 358/950 (37.7%), None 23/950 (2.4%); and LPP—CT 26/75 (34.7%), CT+ET 34/75 (45.3%), ET 12/75 (16.0%), None 3/75 (4.0%). These patterns support the need to adjust for treatment when estimating subtype effects (Supplementary Table S4 and Figure S3). Overall statistics are presented in Table 2.

### 3.3. Survival Analysis

#### 3.3.1. Univariate Survival Analysis

**Figure 3:**
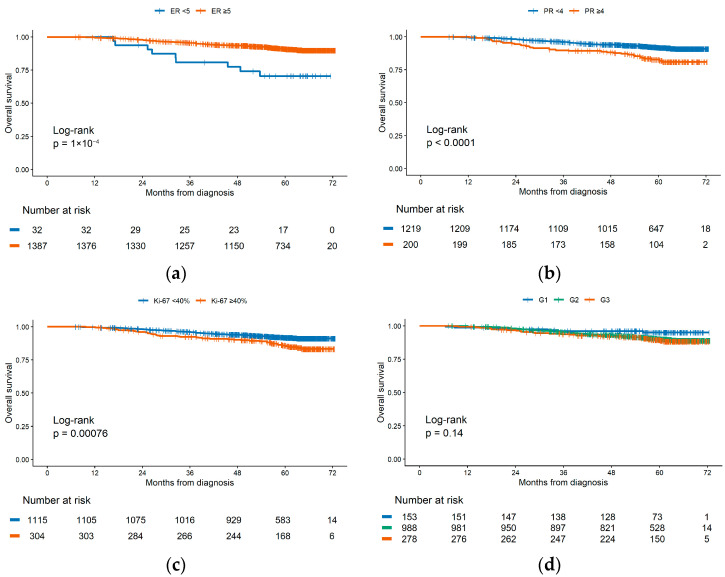
Kaplan–Meier overall survival curves in the HR+/HER2− stage I–III cohort based on ER, PR, Ki-67, and grade status. (**a**) Overall survival by ER status dichotomized at Allred <5 vs. ≥5; (**b**) overall survival by PR status dichotomized at Allred <4 and ≥4; (**c**) overall survival by Ki-67 dichotomized at ≥40% and <40% levels; (**d**) overall survival by grade status divided into G1, G2, and G3 groups. Overall survival (OS) was defined as time from diagnosis to death.

In the HR+/HER2− stage I–III cohort (*n* = 1419; 130 deaths), we compared OS after dichotomizing ER as <5 vs. ≥5 (Allred) (Figure 3a) and PR as <4 vs. ≥4 (Figure 3b), and additionally examined Ki-67 (≥40% vs. <40%) (Figure 3c) and histologic grade (Figure 3d). Median OS was not reached in any stratum. For PR, patients with PR <4 had significantly higher mortality than those with PR ≥ 4 (HR 2.25, 95% CI 1.52–3.33); the 60-month OS was 82.6% vs. 91.8%. For Ki-67, the ≥40% group had worse outcomes than the <40% group (HR 1.85, 1.29–2.67; 60-month OS 85.8% vs. 91.8%). For grade, hazards were higher—though not statistically significant—for G2 (HR 2.03, 0.94–4.38) and G3 (HR 2.26, 0.99–5.17) relative to G1; corresponding 60-month OS estimates were 90.1% (G2) and 89.4% (G3) vs. 95.0% (G1). When ER was dichotomized as <5 vs. ≥5, the ER < 5 group was associated with significantly worse survival (HR = 3.52; 95% CI [1.79–6.93]; *p* < 0.001) compared to the ≥5 group. Taken together, PR ≥ 4 and Ki-67 ≥ 40% identify subgroups with clearly inferior OS, whereas grade shows a non-significant gradient and ER < 5 vs. ≥ 5 does not materially discriminate risk in this dataset.

#### 3.3.2. «Luminal B Poor-Prognosis» Subgroup Identification

To ensure reproducibility, all HR+/HER2− tumors were classified using a fixed rule set. The LPP subset consisted exclusively of tumors with PR < 4 (Allred) and Ki-67 ≥ 40%. All other HR+/HER2− tumors were categorized as Luminal A or Luminal B based on standard immunohistochemical criteria. Discordant patterns (e.g., low PR but moderate Ki-67, or high Ki-67 with preserved PR) were assigned to Luminal B according to predefined rules (see Methods Section 2.8).

**Figure 4:**
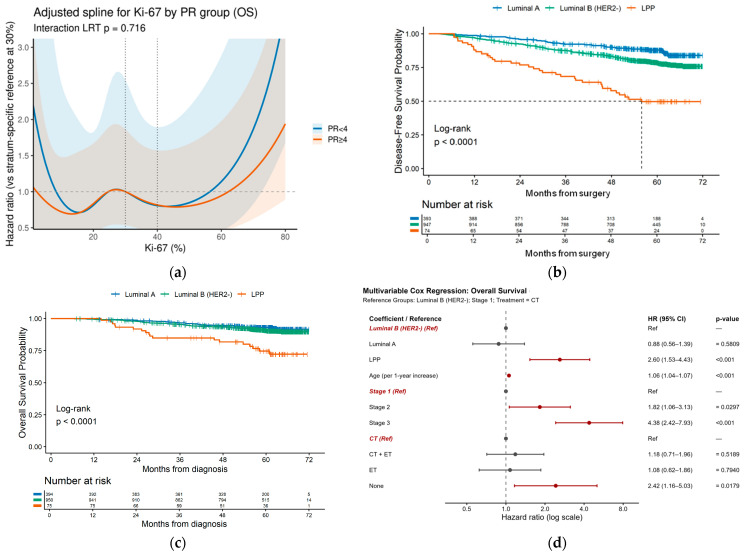
Identification and prognostic impact of the “LPP” subgroup in HR+/HER2− breast cancer. (**a**) Penalized spline of overall survival by Ki-67 and PR. The horizontal dashed line at HR = 1 denotes no difference in hazard relative to the stratum-specific reference. For each PR stratum (blue: PR < 4; orange: PR ≥ 4), the spline curve is centered at Ki-67 = 30%, so HR=1 corresponds to the hazard at Ki-67 = 30% within that PR group. The vertical dashed lines at 30% and 40% mark the two pre-specified candidate Ki-67 cutoffs evaluated in the study; they are visual guides only and were not used to fit the spline. Shaded bands show the 95% confidence intervals; (**b**) disease-free survival for LPP, Luminal B, and Luminal A. Dashed line represents 50% disease-free survival (DFS) probability; (**c**) Kaplan–Meier overall survival by subgroup; (**d**) multivariable Cox regression (forest plot) for survival predictors. Statistically significant predictors are highlighted with red. Dashed line represents hazard ratios (HR) = 1.0. Overall survival (OS) was defined as time from diagnosis to death. For localized disease (stages I–III), DFS was defined as time from curative surgery to relapse or death. Relapse events were identified from EMR text using the earliest exact date (dd.mm.yyyy); uncertain dates were ignored. Patients without a dated relapse were censored at last visit.

For studying the relationship between Ki-67 levels and overall survival (OS) risk function, spline curves were used to smooth data and visually demonstrate how different Ki-67 levels affect death risk. Spline curves were flat across 20–35% and increased beyond ~35–40%, with a consistently higher trajectory in PR < 4 (Figure 4a). Visually, the hazard function for OS stays low and flat up to ~30–35% Ki-67 and then rises sharply, particularly in PR-negative tumors. However, formal interaction testing did not reach significance (*p* = 0.716), so we did not find strong evidence that the Ki-67 effect slope differs by PR status. These findings support using 40% as a clinically anchored “very-high” Ki-67 threshold and, together with PR < 4, define a high-risk “LPP” subset.

To evaluate whether the prognostic signal was unique to the dual “double-hit” phenotype or was already captured by looser definitions, we performed sensitivity analysis across four rule sets (Supplementary Table S3). Each individual marker was associated with inferior OS. For example, low PR (<4) vs. ≥4 showed HR 2.25 (95% CI 1.52–3.33; *p* < 0.001; LR χ^2^ 14.30; c-index 0.564). For Ki-67, high values (≥40%) vs. <40% yielded HR 1.85 (1.29–2.67; *p* < 0.001; LR χ^2^ 10.12; c-index 0.556).

A broader “high-risk luminal” OR-rule (PR < 4 or Ki-67 ≥ 40%) also showed significant separation (HR 1.94, 1.38–2.75; *p* < 0.001; LR χ^2^ 13.73; c-index 0.574), indicating that either abnormality alone contributes to risk.

However, the double-hit LPP rule (PR < 4 and Ki-67 ≥ 40%) produced the largest effect size (HR 3.25, 1.98–5.35; *p* < 0.001), the strongest likelihood ratio improvement, and the lowest AIC (1827.4 vs. 1829.3–1833.5 for alternative rules), despite comprising the smallest subset (*n* = 75; 18 events).

Thus, a looser OR-rule identifies a general risk gradient, but the double-hit phenotype uniquely concentrates the highest-risk biology, yielding superior prognostic discrimination.

From the final dataset, we identified HR+/HER2− tumors as Luminal A, Luminal B (HER2−), which was characterized by PR ≥4 and Ki-67 < 40%, or LPP based on prespecified rule from standard IHC (PR <4 on Allred and Ki-67 ≥40%). DFS differed significantly between the three cohorts (global log-rank *p* < 0.001; Figure 4b) and was directionally concordant with OS. Pairwise comparisons in the log-rank test (Benjamin–Hochberg correction) showed Luminal B to do worse than Luminal A (*p* < 0.001), while LPP did worse than Luminal A (*p* < 0.001) and Luminal B (*p* < 0.001). Univariable Cox models, with Luminal A as a reference group, showed that hazard ratio (HR) for progression was HR 1.69 (95% CI 1.24–2.29, *p* < 0.001) for Luminal B and HR 4.84 (3.17–7.41, *p* < 0.001) for LPP, and the model showed limited discriminative ability with a c-index of 0.587. Overall survival analyses showed similar ordering, with steep separation of curves and significantly poorer outcome in LPP (*p* < 0.001; Figure 4c).

Overall, the best-performing rule among candidates under all criteria was the two-marker rule “PR < 4 and Ki-67 ≥ 40%”. Under OS, it generated the greatest effect size among definition tests (HR 3.25, 95% CI 1.98–5.35) with practical prevalence of 5.3% (75/1419). The inclusion of grade 3 reduced prevalence to 2.6% and failed to improve performance (HR 3.14, poorer concordance); Ki-67 ≥ 40% plus grade 3 without PR failed to reach significance. Additionally, model discrimination and fit were both weaker in this group (concordance index 0.524; LR-χ^2^ = 8.02, df = 1, *p* = 0.005). Of note, 60-month OS was 77.0% for this subset versus 90.9% for the rest (Supplementary Figures S4 and S5).

To determine the independent prognostic utility of the luminal poor-prognosis (LPP) subtype within the HR+/HER2− cohort, a multivariable Cox proportional hazards regression model was performed for Overall Survival (OS). The final model included the molecular subtypes (LPP, Luminal A, and Luminal B (HER2−)), along with the essential clinical covariates: age at diagnosis (continuous), AJCC Stage (stage I as reference), and Combined Treatment Regimen (CT as reference).

The final model exhibited robust discriminatory power, with a C-index of 0.766. This represents a measurable improvement in model fit compared to the baseline clinical model (age, stage, and treatment only; C-index= 0.753), affirming the incremental prognostic value of integrating the molecular subtype classification. In the fully adjusted analysis, the LPP subtype was confirmed to be a potent and independent factor associated with an adverse prognosis. Using the refined Luminal B (HER2−) group (excluding LPP cases) as the reference, patients classified as LPP demonstrated a 2.60-fold increased risk of mortality (HR 2.60; 95% CI: 1.53–4.43; *p* = 0.00042). This finding demonstrates that the adverse outcome of LPP tumors is independent of established clinical variables and confounding treatment differences (Figure 4d).

As result, all established clinical prognostic factors included in the model retained high statistical significance. Age at diagnosis was significantly associated with OS, with the risk of death increasing by 5.6% for every one-year increment in age (HR: 1.06; 95% CI: 1.04–1.08; *p* < 0.001). Similarly, stage classification was confirmed as the strongest prognostic determinant. Compared to stage I, stage III disease was associated with a 4.38-fold increased risk of death (HR: 4.38; 95% CI: 2.42–7.93; *p* < 0.001), while stage II disease showed a 1.82-fold increase (HR: 1.82; 95% CI: 1.06–3.14; *p* = 0.0297). As for treatment regimen, when compared to the reference regimen (CT), patients who received no adjuvant systemic therapy (None) demonstrated a 2.42-fold higher risk of death (HR: 2.42; 95% CI: 1.16–5.03; *p* = 0.0179).

## 4. Discussion

### 4.1. LLM Performance in Data Extraction

This study showed that LLM Claude Sonnet 3.7 was able to extract breast cancer variables from Russian-language EMRs with a high degree of accuracy. In keeping with recent work validating LLMs for pathology report parsing, the concordance with human annotation for ER, PR, HER2, and grade was considerably high [32]. While Ki-67 values demonstrated strong reliability (ICC ≈ 0.88) with sporadic outliers around therapeutic thresholds (20–30%), HER2 and receptor score extraction was particularly robust. The need for human oversight in borderline cases is highlighted by the fact that this reflects known interobserver variability in Ki-67 scoring [33,34].

Extracting temporal data, like progression dates, was more difficult; when both AI and humans recorded values, there could be differences of up to years, and “agreement” was frequently based on joint missingness. This illustrates the persistent challenges in extracting temporal data from medical records [35,36]. When combined, our findings show that LLMs are capable of accurately structuring static clinicopathologic data, but they are less reliable when it comes to longitudinal variables. Crucially, our results support growing evidence that LLMs continue to perform well in healthcare data that is not in English, despite the lack of systematic multilingual benchmarks. Importantly, the clinical outcome patterns we observed (e.g., impact of Ki-67 and PR) were consistent with known prognostic data, suggesting that the data quality was still sufficient to yield valid conclusions despite the noise in extracted dates.

### 4.2. Clinical Findings and the LPP Subtype

The prognostic significance of PR and Ki-67 was confirmed by analyzing 1419 stage I–III HR+/HER2− patients. While ER thresholds did not significantly stratify risk, low PR (<4) was associated with significantly worse survival (HR = 2.25) and high Ki-67 (≥40%) likewise conferred worse outcomes (HR = 1.85). There was a non-significant negative gradient in histologic grade.

A distinct “LPP” subgroup (5.3% prevalence) was defined by a composite phenotype: PR < 4 and Ki-67 ≥ 40%. Compared to Luminal A and Luminal B, this group’s results were noticeably worse (HR for progression vs. Luminal A 4.84; fully adjusted OS HR 2.60 [95% CI: 1.53–4.43], *p* = 0.00042). Interestingly, following the separation of LPP, Luminal A showed no statistically significant differences from standard Luminal B (HER2−) (HR 0.88, *p* = 0.5809), suggesting that the poor prognosis in HR+/HER2− disease might be influenced by LPP phenotype. This suggestion was supported by statistically significant difference between LPP vs. the Luminal B phenotype, confirming that LPP is distinctly higher-risk than Luminal B after excluding LPP from that group. However, these findings are preliminary and require further validation on more balanced data. Our observation that the LPP subtype retains strong prognostic significance even after adjustment for the treatment regimen may be particularly important. This is consistent with data suggesting that the independent prognostic value of Ki-67 alone often shows inconsistency in multivariate models adjusted for clinical factors and therapy [37,38]. This may indicate that combining high Ki-67 with PR loss provides more robust risk stratification.

Our findings support previous research showing that elevated Ki-67 and PR loss are independent predictors of adverse outcomes [39,40]. International guidelines already use these markers to distinguish between Luminal B-like tumors and Luminal A-like tumors (low Ki-67, high PR). However, the prognosis varies because many Luminal B tumors have only one unfavorable feature. By distinguishing the “ultra-high risk” group from the other luminal disease groups, our definition of LPP—PR loss plus very high Ki-67—improves risk stratification. For instance, TEXT and SOFT trials have shown that exemestane plus ovarian suppression provides a greater absolute benefit to premenopausal women with HR-positive, HER2-negative early breast cancer who have low PR and/or high Ki-67, although each marker by itself has limited predictive value for adjuvant endocrine selection [41]. Additionally, 2021St. Gallen/Vienna Consensus suggested a high Ki-67 as a high-risk indicator that favors the addition of adjuvant chemotherapy in early HR+HER2− breast cancer but did not mention any specific PR levels being used for stratification [42]. On the other hand, monarchE showed sustained abemaciclib benefit regardless of Ki-67 status, suggesting that the proliferation index alone is not sufficient to identify who benefits—additional factors like PR loss might improve risk stratification [43].

### 4.3. Limitations

This study has several limitations. It was retrospective, region-specific, and heavily dependent on EMR quality. The raw texts contained substantial missing data, limiting extraction by both LLM and human annotators and reducing dataset completeness. To simplify workflow, we excluded patients with multiple cancers, though such cases exist in clinical practice and would need to be addressed in future models. Due to resource constraints, we did not test different prompting strategies, despite evidence that prompt design can affect LLM outputs. Similarly, validation was suboptimal: while feasible for this initial analysis, a true gold standard would require double-blinded annotation with adjudication by a third expert oncologist. Molecular validation (e.g., PAM50 or more sophisticated analysis) was also lacking, so the proposed “LPP” subtype remains clinicopathologic and hypothetical.

A further major limitation is the lack of detailed treatment annotation. Although we were able to extract a coarse treatment classification (endocrine only, chemotherapy ± endocrine, or no systemic therapy) and include it in adjusted models, granular data such as endocrine therapy type, ovarian suppression, chemotherapy regimen, and duration were not recoverable from EMR text. Because treatment intensity is a major driver of outcomes in HR+/HER2− disease, residual confounding is possible, and the prognostic effect of LPP should be interpreted cautiously until validated in datasets with complete treatment information.

Follow-up was relatively short, limiting long-term prognostic conclusions. Finally, although LLM extraction was accurate for static variables, occasional misclassifications occurred, and temporal data such as progression dates remained unreliable. Therefore, we based all main conclusions on OS, which was reliably captured from structured records, while using the DFS analysis only as supportive evidence, with clearly defined event rules and a brief summary of data completeness. Together, these limitations emphasize the need for more complete source data, robust validation, prompt optimization, and prospective external studies before clinical integration.

### 4.4. Future Directions

At this stage, the LPP signal is hypothesis-generating. Priority next steps are external validation in independent health systems and prospective, treatment-adjusted studies (including decision-impact and escalation/de-escalation trials). Future work should incorporate detailed systemic treatment data, as therapy patterns strongly influence outcomes in HR+/HER2− disease and will help further refine the prognostic role of the LPP phenotype. Clinically, LPP should be validated prospectively and compared with genomic assays (e.g., Oncotype, PAM50) to establish biological distinctiveness. Trials could evaluate whether patients exhibiting this phenotype receive specific advantages from chemotherapy, CDK4/6 inhibitors, or other innovative agents. Simultaneously, identifying Luminal A and Luminal B patients with advantageous profiles may facilitate treatment de-escalation.

Enhancing LLM performance in oncology requires progress in multiple areas. Firstly, hybrid approaches that combine LLMs with probabilistic or rule-based date parsers should be used to improve accurate data extraction, including temporal information. Second, the development of multilingual benchmarks and performing domain-specific fine-tuning on non-English oncology datasets will enhance generalizability and accuracy. Lastly, clinical trust in medical LLMs will depend on improving robustness and transparency through adversarial testing, uncertainty quantification, model transparency (explainability), and proper human-in-the-loop validation.

## 5. Conclusions

Our study shows that Russian-language oncology data can be efficiently extracted and formalized using publicly available non-fine-tuned LLMs, enabling in-depth real-world analyses. We identify and describe a hypothetical LPP subgroup of HR+/HER2− breast cancer that might be associated with a significantly worse prognosis than the conventional Luminal A and B subtypes. Being relatively small (5.3%) but clinically meaningful, this subgroup is distinguished by progesterone receptor loss and elevated Ki-67 levels. In addition to generating hypotheses, this work highlights the rising clinical significance of easily accessible, straightforward markers and the transformative potential of LLMs in advancing the role of real-world evidence in precision oncology. However, given the retrospective, single-system design and the absence of treatment adjustment, these findings are hypothesis-generating and warrant external validation and prospective, treatment-adjusted evaluation before informing adjuvant decision-making.

## Figures and Tables

**Table 1:** Major clinical variables extracted from unstructured EMR data used for large language model-based information extraction.

Variable	Expected Format	Examples Supplied in Prompt
Ki-67	numeric (%)	“Ki-67 = 32%” → 32
ER (Allred)	integer 0–8	“ER Allred 7/8” → 7
PR (Allred)	integer 0–8	“PR Allred 5” → 5
HER2 IHC	categorical: 0, 1+, 2+, 3+	“HER2 IHC 3+” → 3+
grade	G1/G2/G3	“grade 2” → G2
local relapse date	dd.mm.yyyy	“local recurrence 14 March 2022”
distant relapse date	dd.mm.yyyy	“bone mts 9 November 2021”
multiple primary cancers	yes/no	prompt rule-based

**Table 2:** Clinicopathological characteristics of the overall cohort and the HR+/HER2− stage I–III sub-cohort. Vital status follow-up was updated through 29 February 2025 via structured EMIAS records.

Characteristics	Overall Cohort (*n* = 2347)	HR+/HER2− Stage I–III Cohort (*n* = 1419)
time of observation	1 January 2019–28 February 2025	
median age at diagnosis	60.8 years (range 23.7–95.0)	61.3 years (range 24.1–99.0)
median follow-up	61.5 months	61.6 months
stage	I—711 (29.9%) II—1015 (43.2%) III—471 (20.2%) IV—150 (6.7%)	I—497 (35.0%) II—631 (44.5%) III—291 (20.5%)
molecular subtypes	Luminal A– 409 (17.4%) Luminal B (HER2−)—1097 (46.7%) Luminal B (HER2+)—698 (29.8%) HR-HER2+—89 (3.8%) Triple negative—54 (2.4%)	Luminal A—394 (27.8%) Luminal B (HER2−)—1025 (72.2%)
median Ki-67	24 (IQR 15–35)	25 (IQR 16–35)
ER	<5—215 (9.1%) ≥5—2132 (90.9%)	<5—32 (2.3%) ≥5—1387 (97.7%)
PR	<4—498 (21.2%) ≥4—1849 (78.8%)	<4—200 (14.1%) ≥4—1219 (85.9%)
grade	G1—201 (8.5%) G2—1575 (67.1%) G3—571 (24.3)	G1—153 (10.8%) G2—988 (69.6%) G3—278 (19.6%)
events	OS (deaths)—317 (13.5%) PFS (relapses)—629 (26.8%)	OS (deaths)—130 (9.2%)

## Data Availability

The data presented in this study are available on request from the corresponding authors due to regulatory and ethical requirement of Moscow Department of Health.

## Supplementary Materials

The following supporting information can be downloaded at: https://www.mdpi.com/article/10.3390/cancers17233836/s1, Table S1: Classification rules for BC molecular phenotypes in HR+HER2- cohort; Table S2: Handling of discordant patterns; Table S3: Sensitivity analysis across various definitions of LPP; Table S4: Treatment regimen distribution in HR+HER2- cohort; Figure S1: Date agreement (rule-based) local and distant progression; Figure S2: Magnitude of date discrepancies (mismatched pairs only); Figure S3: Treatment options distribution in HR+HER2− stage I-III cohort; Figure S4: Survival curves for LPP (Ki67, PR); Figure S5: Survival curves for LPP (Ki67, PR, G3). File S1: an LLM prompt document in Russian

## Abbreviations

The following abbreviations are used in this manuscript:

BC	Breast cancer
CI	Confidence interval
EMR	Electronic medical records
ER	Estrogen receptor
HR	Hazard ratio
HR+/HER2	Hormone-positive HER2-negative
ICC	Intraclass correlation coefficient
IHC	Immunohistochemistry
LLM	Large language model
NAT	Neoadjuvant treatment
AT	Adjuvant treatment
ET	Endocrine therapy
CT	Chemotherapy
LPP	Luminal B poor-prognosis
MAE	Mean absolute error
PR	Progesterone receptor
RMSE	Root mean square error
