# Supplementary material for: Deriving Real-World Evidence from Non-English Electronic Medical Records in Hormone Receptor-Positive Breast Cancer Using Large Language Models
Source: Cancers (Basel). 2025 Nov 29;17(23):3836. doi: 10.3390/cancers17233836 (PMC12691197; doi:10.3390/cancers17233836)
Supplement: Supplementary file 1 [file cancers-17-03836-s001.zip › supplementary file S1.pdf]

Задача

Ты – врач-онколог со специализацией на лечении рака молочной железы (РМЖ). Твоя задача – построчно проанализировать таблицу с описанием случаев рака молочной железы. На основании предоставленных данных необходимо определить клинически значимые параметры.

Тебе даны следующие поля данных:
1. ID пациента (между тегами <ID> и </ID>) .
2. Анамнез заболевания пациента (между тегами <анамнез> и </анамнез>).
3. Патоморфологическое заключение пациента (между тегами <патоморфология> и </патоморфология>).
4. Клинический диагноз пациента (между тегами <диагноз> и </диагноз>).

Инструкция

1. Анализ анамнеза заболевания

1.1 Проанализируй анамнез заболевания пациента. Анамнез заболевания – это история болезни пациента, в которой в хронологическом порядке описано все, что происходило с пациентом с момента начала заболевания до текущего момента. В Анамнезе заболевания указываются жалобы пациента, дата постановка диагноза, лабораторные и инструментальные методы исследования, результаты патоморфологического и молекулярно-генетического исследования, проведенные оперативные вмешательства, проведенное лекарственное лечение и лучевая терапия с датами. Кроме того, в анамнезе заболевания указывается информация о факте прогрессировании онкологического заболевания (локальные рецидивы и отдаленные метастазы), дата прогрессирования, данные об осложнениях от онкологического заболевания или от проводимого по его поводу лечения. Разбери и полностью пойми анамнез заболевания.

1.2 Идентифицируй, какие целевые клинические признаки пациентов из референтного перечня клинических признаков (дан ниже) упоминаются в анамнезе. Процитируй релевантные отрывки текста из исходного поля «анамнез».

2. Анализ патоморфологического заключения пациента

2.1 Проанализируй патоморфологического заключения пациента. Патоморфологическое заключение – это описание макроскопического и микроскопического состояния гистологического материала опухоли после биопсии или после операции. В патоморфологическое заключение может входить окраска гематоксилин-эозином с оценкой основных параметров опухоли - органная принадлежность опухоли, гистологических подтип опухоли, степень злокачественности опухоли и прочее. Также в патоморфологическое заключение может входить информация об иммуногистохимических исследованиях – индекс пролиферативной активности Ki67, рецепторный статус по эстрогенам, рецепторный статус по

прогестерону, рецепторный статус по HER2. Разбери и полностью пойми патоморфологические заключения.

2.2 Идентифицируй ключевые иммуногистохимические маркеры, оцениваемые при раке молочной железы из текста в виде списка. При идентификации используй примеры из референтного списка патоморфологических признаков (дан ниже), но руководствуйся не только ими. Процитируй релевантные отрывки текста из исходного поля «патоморфология».

3. Анализ клинического диагноза заключения пациента

3.1 Проанализируй клинический диагноз пациента. В клинических диагноз пациента может входить, но не ограничиваться этим, информация о органной принадлежности опухоли, ее гистологический подтип, стадия по системе TNM, клиническая стадия, молекулярно-генетический подтип опухоли (Люминальный А, Люминальный В HER2+, Люминальные В HER2-, Нелюминальный HER2+, трижды негативный рак молочной железы). Помимо этого, в клиническом диагнозе может быть указан хронологический порядок всех событий с пациентом в рамках текущего заболевания – от первичной диагностики, результатов лабораторных и инструментальных исследований, проведенного лечения (хирургического, лекарственного, лучевого), прогрессирования и осложнения. Разбери и полностью пойми текст в нем.

3.2 Идентифицируй ключевые признаки, оцениваемые при раке молочной железы из текста в виде списка. При идентификации используй примеры из референтного списка признаков из клинического диагноза (дан ниже), но руководствуйся не только ими. Процитируй релевантные отрывки текста из исходного поля «диагноз».

4. Подготовка ответа

Продумай свой ответ по каждому из следующих полей:
1. «Два_и_более_онкологических_заболевания»
2. «Дата_прогрессирования_локально»
3. «Дата_прогрессирования_отдаленно»
4. «Ki67»
5. «Grade»
6. «ER»
7. «PR»
8. «HER2»

Сначала приведи обоснование, затем сформулируй ответ. Руководствуйся логикой и примерами из референтных перечней. приведи цитаты исходного текста, где необходимо.

5. Синтез ответа

Синтезируй свой ответ по следующему шаблону из примера (полный пример дан между тегами <пример> и </пример>, пример ответа дан между тегами <ответ> и </ответ> внутри тегов <пример> и </пример>):

<пример>
<задача>
<ID>
…
</ID>

<анамнез>
…
</анамнез>

<патоморфология>
…
</патоморфология>

<диагноз>
…
</диагноз>
</задача>

<ответ>
1. <Два_и_более_онкологических_заболевания>
Нет
</Два_и_более_онкологических_заболевания>

2. <Дата_прогрессирования_локально>
07.2020
</Дата_прогрессирования_локально>

3. <Дата_прогрессирования_отдаленно>
07.2020
</Дата_прогрессирования_отдаленно>

4. <Ki67>
95%
</Ki67>

5. <Grade>
G3
</Grade>

6. <ER>
-
</ER>

7. <PR>
-
</PR>

8. <HER2>
HER2 -
</HER2>

</ответ>
</пример>

Четко следуй шаблону без каких-либо отклонений от заданного формата. Обязательно обрами свой финальный ответ тегами <ответ> и </ответ>.

Руководство
- Думай вслух. Записывай свои мысли на каждом этапе выполнения инструкции. При этом твои рассуждения не должны превышать по объему 6 листов А4.
- В некоторых случаях данные в исходных полях могут противоречить друг другу. Если данные противоречат друг другу – выбирай данные из поля «анамнез» как истинные и используй их в дальнейшем анализе.
- Если у пациента два и более онкологических заболеваний, заполни только первое поле «Два_и_более_онкологических_заболевания», в остальных полях поставь прочерк.
- При поиске даты прогрессирования (локальный рецидив и отдаленные метастазы) при отсутствии данных о прогрессировании оставлять поле пустым в ответе, а при нахождении даты прогрессирования – заполнять поле в формате dd.mm.yyyy или mm.yyyy. Если в тексте дата обнаружения локального рецидива и отдаленных метастазов одинаковая, то оба поля заполняются одним и тем же значением.

Референтный перечень клинических признаков

1. Два_и_более_онкологических_заболевания – статус пациента в аспекте наличия двух и более любых онкологических заболеваний. Это могут быть одновременно возникшие онкологические заболевания – рак левой молочной железы и правой молочной железы. Это могут быть онкологические заболевания, возникшие в разное время – рак левой молочной железы и меланомы кожи спины. Указывается это в тексте в виде как минимум двух онкологических диагнозов в текстовом формате. Примеры формулировок: «В 2019 году диагностирован первично-синхронный рак левой и правой молочной железы», «В 2019 диагностирован рак левой молочной железы, в 2021 год выявлена меланома кожи спины». В пограничных случаях отвечай «Да». Наличие доброкачественных новообразований не влияет на данный признак.

2. Дата_прогрессирования_локально – дата, в которую у пациента с онкологическим заболеванием после проведенного лечения впервые был зафиксирован возврат заболевания локально. Дата может быть указана в численном формате (пример: 01.01.2022) или смешанном формате (пример: апрель 2024 года). Примеры

формулировок: «прогрессирование от 21.01.2021», «локальный рецидив 20.08.2023», «рецидив в марте 2020», «продолженный рост от 21.01.2021».

3. Дата_прогрессирования_отдаленно – дата, в которую у пациента с онкологическим заболеванием после проведенного лечения впервые был зафиксирован возврат заболевания в других органах и тканях (легкие, печень, кости, головной мозги и прочее). Дата может быть указана в численном формате (пример: 01.01.2022) или смешанном формате (пример: апрель 2024 года). Примеры формулировок: «прогрессирование от 21.01.2021», «прогрессирование заболевания 20.08.2023», «выявлены новые очаги в марте 2020», «в исследовании от 03.07.2024 определяются метастазы».

4. Ki67 – это численный показатель, измеряемый в процентах при иммуногистохимическом исследовании опухоли молочной железы, который отражает степень пролиферативной активности опухоли. Пример «Ki67 35%», «Индекс пролиферативной активности Ki67% = 75%», «Ki67 равен 14%», «Индекс Ki67 = 29 процентов». Данный показатель может иметь значения только в пределах от 0% до 100%.

5. Grade – это показатель степени злокачественности опухоли на основе балльной Ноттингемской шкалы. Всего существует 3 степени злокачественности: Grade 1 (G1), Grade 2 (G2) и Grade 3 (G3). В оценку входят оценка формирования железистых структур (1-3 балла, чем больше баллов – тем меньше опухоль похожа на нормальную), оценка количества митозов (1-3 баллов, чем больше баллов - тем больше количество митозов), ядерный полиморфизм (1-3 баллов, чем больше баллов – тем больше разнородность ядер в клетках). В итоге по сумме баллов выставляется Grade (G, степень) злокачественности: Grade 1 (G1, Грейд 1, степень злокачественности 1) при 3-5 баллах, Grade 2 (G2, Грейд 2, степень злокачественности 2) при 6-7 баллах, Grade 3 (G3, Грейд 3, степень злокачественности 3) при 8-9 баллах. Примеры: «Степень злокачественности G2», «Степень злокачественности Grade 2», «Степень злокачественности 3», «Степень дифференцировки G1», «Grade 3», «G3».

6. ER – это численный показатель уровня экспрессии (степени представленности) рецепторов эстрогена на поверхности опухолевых клеток при иммуногистохимическом исследовании по системе Allred в баллах. Числовое значение может быть только в диапазоне от 0 до 8 баллов, где 0 баллов - полное отсутствие рецепторов, а 8 баллов - все клетки имеют эстрогеновые рецепторы. Примеры: «ЭР 8 баллов», «Эстрогеновые рецепторы 4 балла», «ER = 6 баллов по Allred», «ЭР=3 балла», «ER 8 баллов», «Рецепторы эстрогена 0 баллов», «ER-».

7. PR – это численный показатель уровня экспрессии (степени представленности) рецепторов прогестерона на поверхности опухолевых клеток при иммуногистохимическом исследовании по системе Allred в баллах. Числовое значение может быть только в диапазоне от 0 до 8 баллов, где 0 баллов - полное отсутствие рецепторов, а 8 баллов - все клетки имеют прогестероновые рецепторы. Примеры: «ПР 8 баллов», «Прогестероновые рецепторы 4 балла», «PR = 6 баллов по Allred», «ПР=3 балла», «PR 8 баллов», «PgR 8», «Рецепторы прогестерона 0 баллов», «PR-».

8. HER2 – это численный показатель уровня экспрессии (степени представленности) рецепторов HER2 на поверхности опухолевых клеток при иммуногистохимическом исследовании. Выделяется 4 варианта HER2 статуса – HER2-отрицательный (HER2-, HER2 0+), HER2 1+, HER2 2+ FISH - (FISH отрицательный), HER2+ FISH+ (FISH положительный), HER2 3+. При вариантах HER2-отрицательный (HER2-, HER2 0+), HER2 1+, HER2 2+ FISH - (FISH отрицательный) – опухоль считается HER2-негативной. При HER2+ FISH+ (FISH положительный), HER2 3+ опухоль считается HER2-позитивной. Примеры: «HER2 - (0)», «HER2 - 1+», «HER2 - 2+ FISH-», «HER2 - 3+», «HER2 - не обнаружено», «HER2 - отрицательный», «HER2 - положительный», «HER2 - негативный», «1+», «2+», «3+», «HER2-».

Референтный перечень патоморфологических признаков

1. Индекс Ki67 – это численный показатель, измеряемый в процентах при иммуногистохимическом исследовании опухоли молочной железы, который отражает степень пролиферативной активности опухоли. Пример «Ki67 35%», «Индекс пролиферативной активности Ki67% = 75%», «Ki67 равен 14%», «Индекс Ki67 = 29 процентов». Данный показатель может иметь значения только в пределах от 0% до 100%.

2. Степень злокачественности (G, Grade) – это показатель степени злокачественности опухоли на основе балльной Ноттингемской шкалы. Всего существует 3 степени злокачественности: Grade 1 (G1), Grade 2 (G2) и Grade 3 (G3). В оценку входят оценка формирования железистых структур (1-3 балла, чем больше баллов – тем меньше опухоль похожа на нормальную), оценка количества митозов (1-3 баллов, чем больше баллов - тем больше количество митозов), ядерный полиморфизм (1-3 баллов, чем больше баллов – тем больше разнородность ядер в клетках). В итоге по сумме баллов выставляется Grade (G, степень) злокачественности: Grade 1 (G1, Грейд 1, степень злокачественности 1) при 3-5 баллах, Grade 2 (G2, Грейд 2, степень злокачественности 2) при 6-7 баллах, Grade 3 (G3, Грейд 3, степень злокачественности 3) при 8-9 баллах. Примеры: «Степень злокачественности G2», «Степень злокачественности Grade 2», «Степень злокачественности 3», «Степень дифференцировки G1», «Grade 3», «G3».

3. Рецепторный статус по эстрогеновым рецепторам – это численный показатель уровня экспрессии (степени представленности) рецепторов эстрогена на поверхности опухолевых клеток при иммуногистохимическом исследовании по системе Allred в баллах. Числовое значение может быть только в диапазоне от 0 до 8 баллов, где 0 баллов - полное отсутствие рецепторов, а 8 баллов - все клетки имеют эстрогеновые рецепторы. Примеры: «ЭР 8 баллов», «Эстрогеновые рецепторы 4 балла», «ER = 6 баллов по Allred», «ЭР=3 балла», «ER 8 баллов», «Рецепторы эстрогена 0 баллов».

4. Рецепторный статус по прогестероновым рецепторам – это численный показатель уровня экспрессии (степени представленности) рецепторов прогестерона на поверхности опухолевых клеток при иммуногистохимическом исследовании по системе Allred в баллах. Числовое значение может быть только в диапазоне от 0 до 8 баллов, где 0 баллов - полное отсутствие рецепторов, а 8 баллов - все клетки имеют

прогестероновые рецепторы. Примеры: «ПР 8 баллов», «Прогестероновые рецепторы 4 балла», «PR = 6 баллов по Allred», «ПР=3 балла», «PR 8 баллов», «PgR 8», «Рецепторы прогестерона 0 баллов».

5. Рецепторный статус по HER2 рецептору – то численный показатель уровня экспрессии (степени представленности) рецепторов HER2 на поверхности опухолевых клеток при иммуногистохимическом исследовании. Выделяется 4 варианта HER2 статуса – HER2-отрицательный (HER2-, HER2 0+), HER2 1+, HER2 2+ FISH - (FISH отрицательный), HER2+ FISH+ (FISH положительный), HER2 3+. При вариантах HER2-отрицательный (HER2-, HER2 0+), HER2 1+, HER2 2+ FISH - (FISH отрицательный) – опухоль считается HER2-негативной. При HER2+ FISH+ (FISH положительный), HER2 3+ опухоль считается HER2-позитивной. Примеры: «HER2 - (0)», «HER2 - 1+», «HER2 - 2+ FISH-», «HER2 - 3+», «HER2 - не обнаружено», «HER2 - отрицательный», «HER2 - положительный», «HER2 - негативный», «1+», «2+», «3+».

Референтный перечень признаков в клиническом диагнозе

1. Два_и_более_онкологических_заболевания – статус пациента в аспекте наличия двух и более любых онкологических заболеваний. Это могут быть одновременно возникшие онкологические заболевания – рак левой молочной железы и правой молочной железы. Это могут быть онкологические заболевания, возникшие в разное время – рак левой молочной железы и меланомы кожи спины. Указывается это в тексте в виде как минимум двух онкологических диагнозов в текстовом формате. Примеры формулировок: «В 2019 году диагностирован первично-синхронный рак левой и правой молочной железы», «В 2019 диагностирован рак левой молочной железы, в 2021 год выявлена меланома кожи спины». В пограничных случаях отвечай «Да». Наличие доброкачественных новообразований не влияет на данный признак.

2. Дата_прогрессирования_локально – дата, в которую у пациента с онкологическим заболеванием после проведенного лечения впервые был зафиксирован возврат заболевания локально. Дата может быть указана в численном формате (пример: 01.01.2022) или смешанном формате (пример: апрель 2024 года). Примеры формулировок: «прогрессирование от 21.01.2021», «локальный рецидив 20.08.2023», «рецидив в марте 2020», «продолженный рост от 21.01.2021».

3. Дата_прогрессирования_отдаленно – дата, в которую у пациента с онкологическим заболеванием после проведенного лечения впервые был зафиксирован возврат заболевания в других органах и тканях (легкие, печень, кости, головной мозги и прочее). Дата может быть указана в численном формате (пример: 01.01.2022) или смешанном формате (пример: апрель 2024 года). Примеры формулировок: «прогрессирование от 21.01.2021», «прогрессирование заболевания 20.08.2023», «выявлены новые очаги в марте 2020», «в исследовании от 03.07.2024 определяются метастазы».

4. Ki67 – это численный показатель, измеряемый в процентах при иммуногистохимическом исследовании опухоли молочной железы, который отражает степень пролиферативной активности опухоли. Пример «Ki67 35%», «Индекс

пролиферативной активности Ki67% = 75%», «Ki67 равен 14%», «Индекс Ki67 = 29 процентов». Данный показатель может иметь значения только в пределах от 0% до 100%.

5. Grade – это показатель степени злокачественности опухоли на основе балльной Ноттингемской шкалы. Всего существует 3 степени злокачественности: Grade 1 (G1), Grade 2 (G2) и Grade 3 (G3). В оценку входят оценка формирования железистых структур (1-3 балла, чем больше баллов – тем меньше опухоль похожа на нормальную), оценка количества митозов (1-3 баллов, чем больше баллов - тем больше количество митозов), ядерный полиморфизм (1-3 баллов, чем больше баллов – тем больше разнородность ядер в клетках). В итоге по сумме баллов выставляется Grade (G, степень) злокачественности: Grade 1 (G1, Грейд 1, степень злокачественности 1) при 3-5 баллах, Grade 2 (G2, Грейд 2, степень злокачественности 2) при 6-7 баллах, Grade 3 (G3, Грейд 3, степень злокачественности 3) при 8-9 баллах. Примеры: «Степень злокачественности G2», «Степень злокачественности Grade 2», «Степень злокачественности 3», «Степень дифференцировки G1», «Grade 3», «G3».

6. ER – это численный показатель уровня экспрессии (степени представленности) рецепторов эстрогена на поверхности опухолевых клеток при иммуногистохимическом исследовании по системе Allred в баллах. Числовое значение может быть только в диапазоне от 0 до 8 баллов, где 0 баллов - полное отсутствие рецепторов, а 8 баллов - все клетки имеют эстрогеновые рецепторы. Примеры: «ЭР 8 баллов», «Эстрогеновые рецепторы 4 балла», «ER = 6 баллов по Allred», «ЭР=3 балла», «ER 8 баллов», «Рецепторы эстрогена 0 баллов», «ER-».

7. PR – это численный показатель уровня экспрессии (степени представленности) рецепторов прогестерона на поверхности опухолевых клеток при иммуногистохимическом исследовании по системе Allred в баллах. Числовое значение может быть только в диапазоне от 0 до 8 баллов, где 0 баллов - полное отсутствие рецепторов, а 8 баллов - все клетки имеют прогестероновые рецепторы. Примеры: «ПР 8 баллов», «Прогестероновые рецепторы 4 балла», «PR = 6 баллов по Allred», «ПР=3 балла», «PR 8 баллов», «PgR 8», «Рецепторы прогестерона 0 баллов», «PR-».

8. HER2 – это численный показатель уровня экспрессии (степени представленности) рецепторов HER2 на поверхности опухолевых клеток при иммуногистохимическом исследовании. Выделяется 4 варианта HER2 статуса – HER2-отрицательный (HER2-, HER2 0+), HER2 1+, HER2 2+ FISH - (FISH отрицательный), HER2+ FISH+ (FISH положительный), HER2 3+. При вариантах HER2-отрицательный (HER2-, HER2 0+), HER2 1+, HER2 2+ FISH - (FISH отрицательный) – опухоль считается HER2-негативной. При HER2+ FISH+ (FISH положительный), HER2 3+ опухоль считается HER2-позитивной. Примеры: «HER2 - (0)», «HER2 - 1+», «HER2 - 2+ FISH-», «HER2 - 3+», «HER2 - не обнаружено», «HER2 - отрицательный», «HER2 - положительный», «HER2 - негативный», «1+», «2+», «3+», «HER2-».

Примеры логики поиска целевых показателей в сырых данных (один вид рака у пациента)

1. Чтение текста в столбце «Анамнез» выявило следующую информацию: «В апреле 2019 году выявлен рак правой и левой молочной железы. Гист.ан. правой молочной железы- инвазивный дольковый рак, ЭР -+8, Пр - 7+ HER 2 - 0. Гистологическое заключение левой молочной железы + ИГХ: - ЭР -8, Пр - 8+ HER/2 - 0. С мая по 02.09.19 в ЛРЦ - проведено 4 курса НАПХТ. С 23.09.19г проведено 8 курсов ХТ таксанами (Паклитаксел). С 24.02. по 02.03.20г находилась на стац лечении в ЛРЦ, проведено хирургическое лечение: РМЭ ЛМЖ. С 23.03. по 30.03.20г находилась на стац лечении в ЛРЦ, проведен второй этап хирургического лечения: РМЭ ПМЖ с пластикой подмышечно-подключично-подлопаточной области композитным мышечным трансплантатом. Гистология: инвазивный дольковый рак. ИГХ: ЭР-8б, РП-7б, Her-2/neu 1, Ki67-5%.Планируется ДЛТ. В период с 23.06.2020г по 31.07.2020г проведен курс ДЛТ СОД 50 Гр, РОД 2Гр; В апреле 2019 году выявлен рак правой и левой молочной железы. Гист.ан. правой молочной железы- инвазивный дольковый рак, ЭР -+8, Пр - 7+ HER 2 - 0. Гистологическое заключение левой молочной железы + ИГХ: - ЭР -8, Пр - 8+ HER/2 - 0. С мая по 02.09.19 в ЛРЦ - проведено 4 курса НАПХТ. С 23.09.19г проведено 8 курсов ХТ таксанами (Паклитаксел). С 24.02. по 02.03.20г находилась на стац лечении в ЛРЦ, проведено хирургическое лечение: РМЭ ЛМЖ. С 23.03. по 30.03.20г находилась на стац лечении в ЛРЦ, проведен второй этап хирургического лечения: РМЭ ПМЖ с пластикой подмышечно-подключично-подлопаточной области композитным мышечным трансплантатом. Гистология: инвазивный дольковый рак. ИГХ: ЭР-8б, РП-7б, Her-2/neu 1, Ki67-5%.Планируется ДЛТ. В период с 23.06.2020г по 31.07.2020г проведен курс ДЛТ СОД 50 Гр, РОД 2Гр Пациентка от гормонотерапии категорически отказалась (в анамнезе остеопения, тромбоз глуб. вен н/кон);». В тексте указано «В апреле 2019 году выявлен рак правой и левой молочной железы. Гист.ан. правой молочной железы- инвазивный дольковый рак, ЭР -+8, Пр - 7+ HER 2 - 0. Гистологическое заключение левой молочной железы + ИГХ: - ЭР -8, Пр - 8+ HER/2 - 0.», что однозначно свидетельствует о наличии 2 видов рака у пациента - рак правой молочной железы и рак левой молочной железы. Соответственно, в столбец «Два_и_более_онкологических_заболевания» вносится значение «Да».

<Два_и_более_онкологических_заболевания>
Да
</Два_и_более_онкологических_заболевания>

2,3. Чтение текста в столбце «Анамнез» выявило следующую информацию: «30.04.20г.- радикальная резекция правой молочной железы с одномоментной аллопластикой кожно-мышечным лоскутом в ГКБ №40. Гистологическое исследование - инвазивный неспецифицированный рак молочной железы 3 степени злокачественности pT2N1aG3. АПХТ не проводилась. Планировалась ДГТ. В ходе обследования перед ДГТ на КТ ОГК от 07.07.20г.- инфильтративное поражение правой молочной железы, очаг в правом легком. ПЭТ КТ от 21.07.20г.- жидкостное образование правой молочной железы и парастернальнойобластисправа». Пациенту была проведена операция, проведено гистологическое исследование послеоперационного материала и проведена компьютерная томография органов грудной клетки (КТ ОГК). По данным КТ от

07.07.2020 было выявлено поражение правой молочной железы и очаг в правом легком. Эти данные свидетельствуют об одновременно выявленном локальном рецидиве (в правой молочной железе) и отдаленном метастазе (очаг в легком). В тексте нет данных о более ранней дате выявленного локального рецидива или отдаленного метастазирования. Соответственно, в поле «Дата_прогрессирования_локально» и поле «Дата_прогрессирования_отдаленно» вносится одинаковое значение «07.07.2020».

<Дата_прогрессирования_локально>
07.07.2020
</Дата_прогрессирования_локально>

<Дата_прогрессирования_отдаленно>
07.07.2020
</Дата_прогрессирования_отдаленно>

4. Чтение текста в столбце «Анамнез» выявило следующую информацию: «На фоне этого в 2021г. выявлен рецидив вобласти лампэктомии правой молочной железы. Выполнено ТАБ новообразования правой молочнойжелезы - рак. Секторальная резекция правой молочной железы 26.11.2021 г в ГКБ №40. Гистологическое исследование, ИГХ № B13719_19 - опухоль in siu, лимфоваскулярная, периневральная инвазия. ЭР-, ПР-, Her2neu - отриц., Ki67 95%. тройной негативный тип». В гистологическом исследовании указано, что у пациентки «Ki67 95%». Указаний в тексте на иные значения нет, поэтому это значение считается корректным. Соответственно, в поле «Ki67» вносится значение «95%».

<Ki67>
95%
</Ki67>

5. Чтение текста в столбце «Анамнез» выявило следующую информацию: «30.04.20г.- радикальная резекция правой молочной железы с одномоментной аллопластикой кожно-мышечным лоскутом в ГКБ №40. Гистологическое исследование - инвазивный неспецифицированный рак молочной железы 3 степени злокачественности pT2N1aG3». В гистологическом исследовании указано «рак молочной железы 3 степени злокачественности pT2N1aG3». Это первое указание на степень злокачественности в тексте, поэтому оно считается корректным. Формулировка «3 степень злокачественности» и «G3» являются эквивалентными. Соответственно, в поле «Grade» вносится значение «G3».

<Grade>
G3
</Grade>

6. Чтение текста в столбце «Анамнез» выявило следующую информацию: «На фоне этого в 2021г. выявлен рецидив вобласти лампэктомии правой молочной железы. Выполнено ТАБ новообразования правой молочнойжелезы - рак. Секторальная

резекция правой молочной железы 26.11.2021 г в ГКБ №40. Гистологическое исследование, ИГХ № B13719_19 - опухоль in siu, лимфоваскулярная, периневральная инвазия. ЭР-, ПР-, Her2neu - отриц., Ki67 95%. тройной негативный тип». В гистологическом исследовании указано, что у пациентки «ЭР-», что является эквивалентом «ER-» и означает отсутствие экспрессии эстрогеновых рецепторов в опухоли молочной железы. Указаний в тексте на иные значения нет, поэтому это значение считается корректным. Соответственно, в поле «ER» вносится значение «ER-».

<ER>
-
</ER>

7. Чтение текста в столбце «Анамнез» выявило следующую информацию: «На фоне этого в 2021г. выявлен рецидив вобласти лампэктомии правой молочной железы. Выполнено ТАБ новообразования правой молочнойжелезы - рак. Секторальная резекция правой молочной железы 26.11.2021 г в ГКБ №40. Гистологическое исследование, ИГХ № B13719_19 - опухоль in siu, лимфоваскулярная, периневральная инвазия. ЭР-, ПР-, Her2neu - отриц., Ki67 95%. тройной негативный тип». В гистологическом исследовании указано, что у пациентки «ПР-», что является эквивалентом «PR-» и означает отсутствие экспрессии прогестероновых рецепторов в опухоли молочной железы. Указаний в тексте на иные значения нет, поэтому это значение считается корректным. Соответственно, в поле «PR» вносится значение «PR-».

<PR>
PR-
</PR>

8. Чтение текста в столбце «Анамнез» выявило следующую информацию: «На фоне этого в 2021г. выявлен рецидив вобласти лампэктомии правой молочной железы. Выполнено ТАБ новообразования правой молочнойжелезы - рак. Секторальная резекция правой молочной железы 26.11.2021 г в ГКБ №40. Гистологическое исследование, ИГХ № B13719_19 - опухоль in siu, лимфоваскулярная, периневральная инвазия. ЭР-, ПР-, Her2neu - отриц., Ki67 95%. тройной негативный тип». В гистологическом исследовании указано, что у пациентки «Her2neu – отриц.», что является эквивалентом «HER2-» и означает отсутствие экспрессии рецепторов HER2 в опухоли молочной железы. Указаний в тексте на иные значения нет, поэтому это значение считается корректным. Соответственно, в поле «HER2» вносится значение «HER2-».

<HER2>
HER2-
</HER2>
