# Supplementary material for: Deriving Real-World Evidence from Non-English Electronic Medical Records in Hormone Receptor-Positive Breast Cancer Using Large Language Models
Source: Cancers (Basel). 2025 Nov 29;17(23):3836. doi: 10.3390/cancers17233836 (PMC12691197; doi:10.3390/cancers17233836)
Supplement: Supplementary file 1 [file cancers-17-03836-s001.zip › supplementary materials.pdf]

cancers MDPI

Supplementary materials of : Deriving Real-World Evidence from Non-English Electronic Medical Records in Hormone Receptor-Positive Breast Cancer Using Large Language Models

Table S1. Classification rules for BC molecular phenotypes in HR+HER2- cohort.

Subtype	Rules
Luminal A (HER2–)	ER ≥ 5 (Allred<br>PR ≥ 4 (Allred) AND<br>Ki-67 ≤ 20%<br>Grade G1-G3<br>HER2 – negative
Luminal B (HER2–)	ER ≥ 5 (Allred)<br>PR < 4 (Allred) OR<br>Ki-67 > 20 and < 40%<br>Grade G1-G3<br>HER2 – negative
LPP (luminal B poor-prognosis)	ER ≥3 (Allred)<br>PR ≤ 4 (Allred) AND<br>Ki-67 ≥ 40%<br>Grade – any<br>HER2 – negative

Table S2. Handling of discordant patterns

Pattern	Classification
PR < 4 AND Ki-67 ≥ 40%	Luminal B poor-prognosis
PR < 4 AND Ki-67 20–39%	Luminal B (HER2–)
PR ≥ 4 AND Ki-67 ≥ 40%	Luminal B (HER2–)
Only one abnormality (PR < 4 OR Ki-67 ≥ 40%)	Luminal B (HER2–)

Table S3. Sensitivity analysis across various definitions of LPP

Comparison	HR (95% CI)	p-value	LR χ^2 (df=1)	c-index	AIC	Group sizes (events)
PR <4 vs PR ≥4	2.25 (1.52–3.33)	<0.001	14.30	0.564	1829.3	166/34 vs 1123/96
Ki-67 ≥40% vs <40%	1.85 (1.29–2.67)	<0.001	10.12	0.556	1833.5	261/43 vs 1028/87
High-risk (PR<4 or Ki≥40) vs Low-risk	1.94 (1.38–2.75)	<0.001	13.73	0.574	1829.9	370/59 vs 919/71
LPP (PR<4 and Ki≥40) vs Non-LPP	3.25 (1.98–5.35)	<0.001	16.23	0.546	1827.4	57/18 vs 1232/112

Table S4. Treatment regimen distribution in HR+HER2- cohort

Subtype	CT	CT+ET	ET	None	Total (n)
**Luminal A**	**49 (12.4%)**	**31 (7.9%)**	**303 (76.9%)**	**11 (2.8%)**	**394**
**Luminal B (HER2−)**	**308 (32.4%)**	**261 (27.5%)**	**358 (37.7%)**	**23 (2.4%)**	**950**
**LPP**	**26 (34.7%)**	**34 (45.3%)**	**12 (16.0%)**	**3 (4.0%)**	**75**

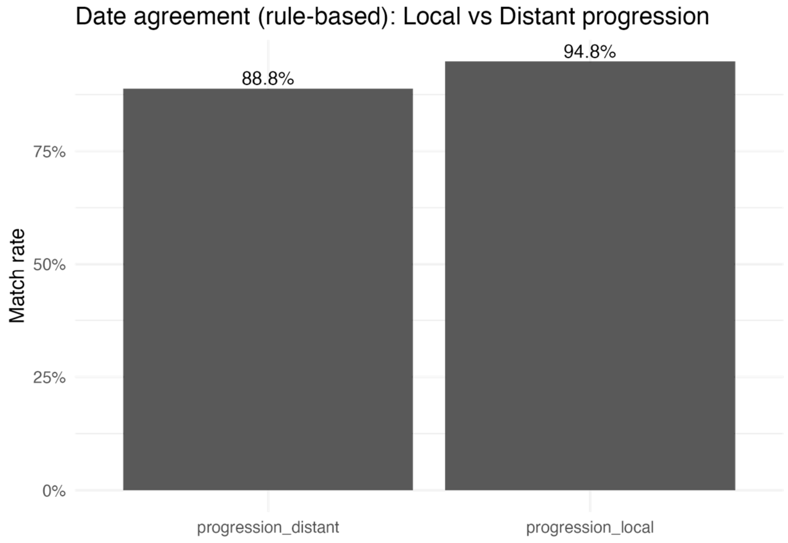

Figure S1. Date agreement (rule-based) local and distant progression.

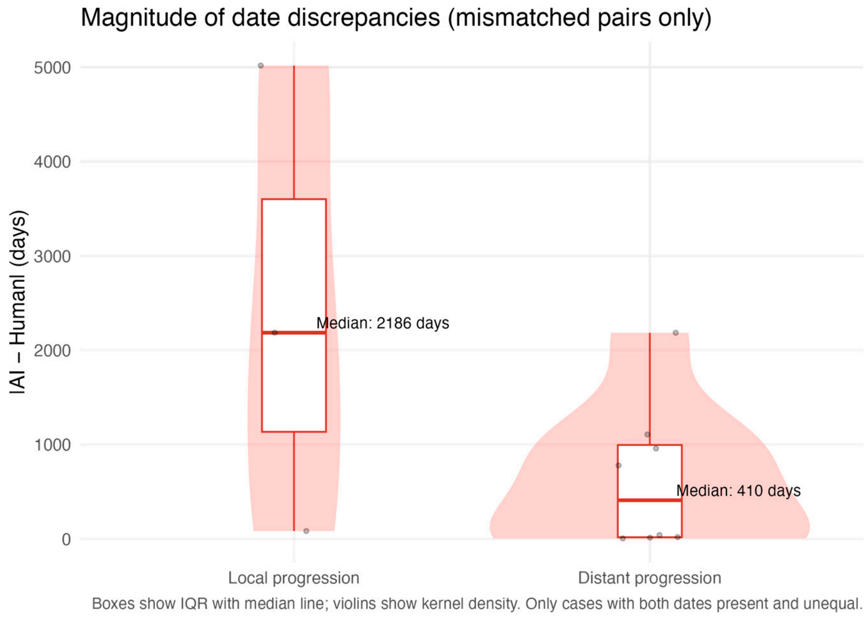

Figure S2. Magnitude of date discrepancies (mismatched pairs only).

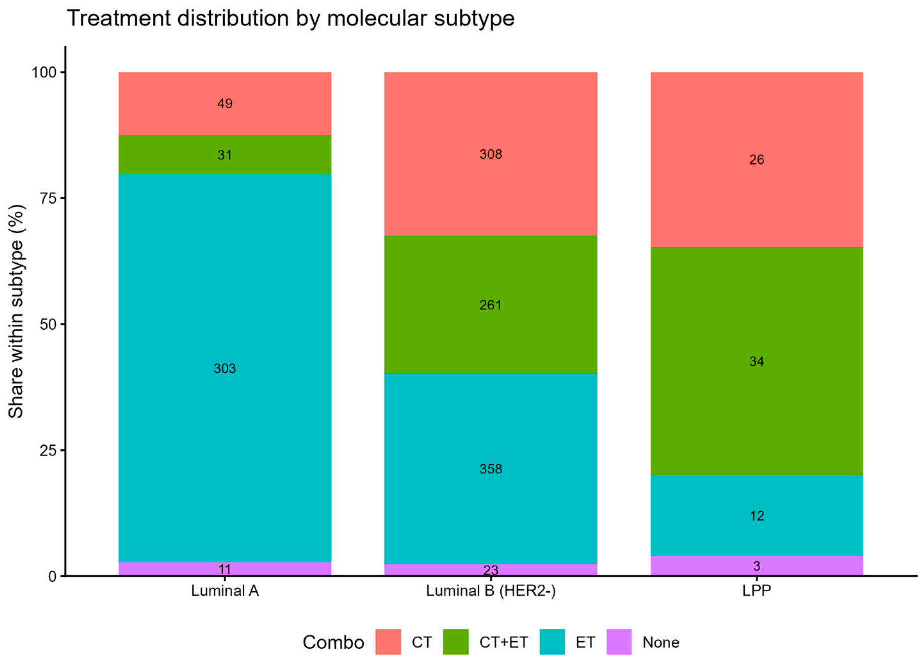

Figure S3. Treatment options distribution in HR+HER2− stage I-III cohort.

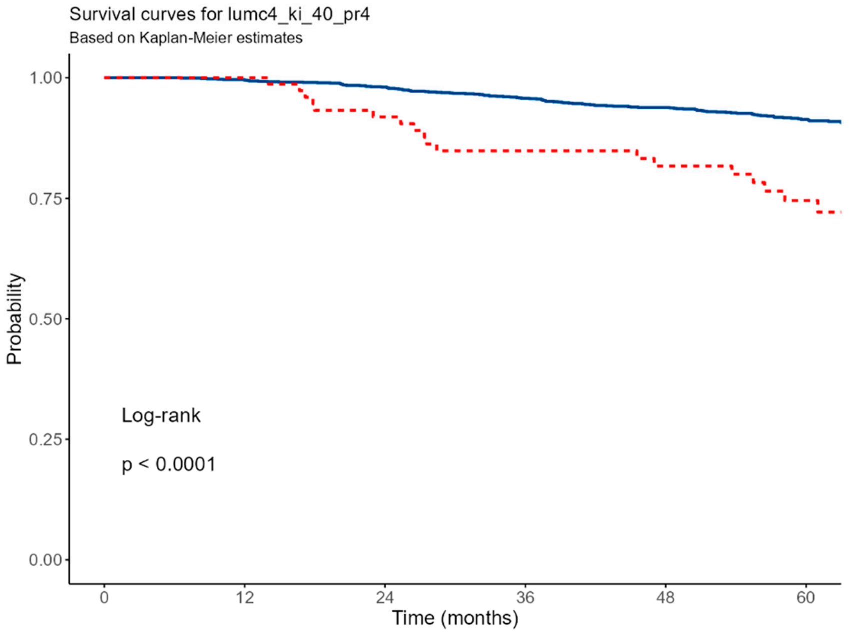

Figure S4. Survival curves for LPP (Ki67, PR).

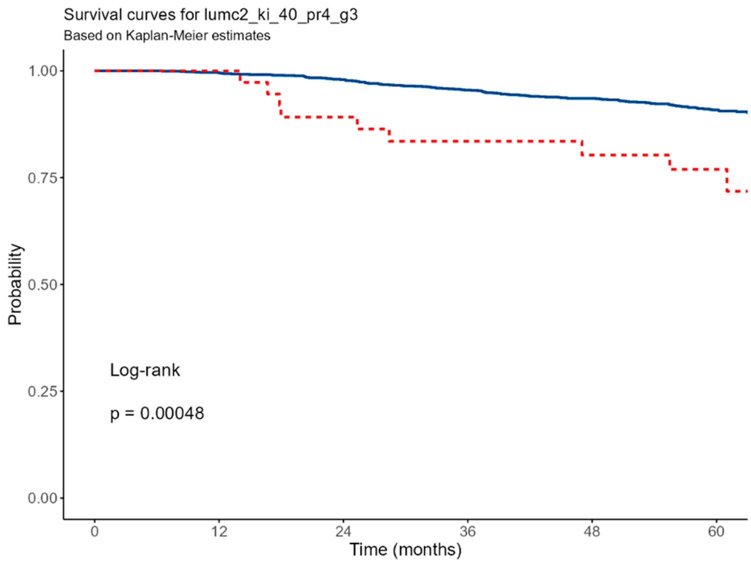

Figure S5. Survival curves for LPP (Ki67, PR, G3).
